# Genome-wide identification and functional validation of RLCK VII subfamily genes conferring disease resistance in broad bean (*Vicia faba* L.)

**DOI:** 10.3389/fpls.2025.1712686

**Published:** 2026-01-21

**Authors:** Fan Wang, Chenwei Liu, Hongchen Lu, Renchao Xu, Xiaochun Bian

**Affiliations:** Key Laboratory of Crop Vernalization Technology in Nantong, Jiangsu Yanjiang Institute of Agricultural Sciences, Nantong, China

**Keywords:** bioinformatics analysis, broad bean, disease resistance function validation, expression characteristic analysis, RLCK VII, VfRLCK VII4

## Abstract

**Introduction:**

Members of receptor like cytoplasmic kinase VII (RLCK VII) subfamily are important participants in plant growth and development, innate immunity, and resistance to abiotic stress. However, in broad beans, the regulatory mechanisms of RLCK VII subfamily genes involved in these processes remains unclear.

**Methods:**

To further elucidate the regulatory mechanisms, a comprehensive whole-genome analysis was conducted. To investigate the disease resistance function of *VfRLCK VII* genes, their expression patterns under infection by *Alternaria alternata* were analyzed through transcriptome sequencing. And functional validation of *VfRLCK VII4 (VfRLCK176)* was performed via transformation into *Nicotiana tabacum* (tobacco).

**Results:**

VfRLCK VII subfamily comprised 45 members, which were unevenly distributed across 6 chromosomes. These genes encoded protein sequences ranging from 296 to 595 aa in length, with 39 located in the nucleus and 6 in chloroplasts. VfRLCK VII proteins were classified into 9 subgroups and 3 members, all of which contained only a single PKc_like superfamily domain. Promoter analysis indicated that *VfRLCK VII* genes possessed various cis-acting elements, including light responsive elements, plant hormone responsive elements, stress responsive elements, and growth and development regulatory elements. Among them, 21 genes exhibited differential expression level, which might be involved in the disease resistance function of broad beans. The disease resistance assessments demonstrated that after inoculation with *A. alternata*, transgenic tobacco plants displayed milder symptoms and significantly smaller lesion areas compared to wild type controls. This finding suggested that VfRLCK VII4 could positively regulate tobacco's resistance to *A. alternata*.

**Discussion:**

This study provides novel insights into the RLCK VII-mediated defense network and offers candidate genes for breeding disease-resistant broad bean varieties.

## 1 Introduction

Receptor like kinases (RLKs) are crucial transmembrane proteins in plants, consisting of an extracellular receptor domain for signal perception, a single transmembrane domain, an intracellular near membrane domain, and cytoplasmic kinase domains that facilitate signal transduction (Liang and Zhou, 2018; Shiu and Bleecker, 2001). Receptor like cytoplasmic kinases (RLCKs) represent a specialized family of protein kinase within the RLK superfamily. They lack both the extracellular receptor domain and the transmembrane domain. Most RLCKs only contain one Ser/Thr intracellular kinase domain, while a few RLCKs also contain other domains such as U-box, leucine rich repeat (LRR), pentapeptide repeat (PPR), WD40, and epidermal growth factor (EGF) (Lehti-Shiu et al., 2009; Li et al., 2002; Vij et al., 2008). In Arabidopsis and rice, a total of 147 AtRLCKs and 379 OsRLCKs have been identified respectively (Shiu et al., 2004; Vij et al., 2008). According to phylogenetic relationships, RLCKs can be divided into 17 subfamilies, including RLCK II, RLCK IV - RLCK XIX, etc (Cen et al., 2023). Early studies have demonstrated that genes in RLCK family are crucial for plant growth and development, signal transduction, and response to biotic and abiotic stresses (Cui et al., 2018; Dubouzet et al., 2011; Muto et al., 2004; Shen et al., 2001).

Among these subfamilies, RLCK VII has been most extensively studied and recognized as a central component of plant immune signaling (Rao et al., 2018). In *Arabidopsis thaliana*, there are 46 members in RLCK VII subfamily, which can be further categorized into 9 subgroups AtRLCK VII-1 - AtRLCK VII-9 and 3 distinct members AtPBL28 (PBS1-like 28), AtPBL29, and AtCDG1 (constitutive differential growth 1) based on protein evolutionary relationships. Among the various subgroups, genes in AtRLCK VII-5, 7, and 8 subgroups are widely involved in pattern recognition receptor (PRR) signal transduction and immune responses, and genes in AtRLCK VII-4 subgroup mediate specific PRR signal transduction and mitogen activated protein kinase (MAPK) activation triggered by chitin (Rao et al., 2018). The most well-characterized member, Botrytis-induced kinase 1 (BIK1), together with PBL1, interacts with receptor kinases such as flagellin-sensing (FLS2), EF-Tu receptor (EFR), chitin elicitor receptor kinase 1 (CERK1) and PEP receptor 1 (PEPR1) to initiate immune responses. These responses include calcium influx, reactive oxygen species (ROS) burst, callose deposition, and stomatal closure (Kadota et al., 2014; Liu et al., 2013; Lu et al., 2010; Zhang et al., 2010). The expression of *OsPBL1* gene in rice can be induced by *Pseudomonas syringae*. And heterologous transformation of the *OsPBL1* gene into Arabidopsis results in increased accumulation of H_2_O_2_ and callus formation in transgenic plants, along with enhanced expression of key genes in the salicylic acid (SA) pathway. This indicates that the *OsPBL1* gene enhances plant disease resistance by activating the SA-dependent pathway (Khare et al., 2016). OsRLCK176 and OsRLCK185, which are homologs of AtBIK1, can mediate immune responses activated by chitin and peptidoglycan through interacting with the cell membrane surface receptor OsCERK1 (Ao et al., 2014; Yamaguchi et al., 2013). In tomato plants, ACIK1 (Avr9/Cf-9 induced kinase 1), a member of RLCK VII subfamily, plays a role in resistance against tomato leaf mold mediated by cell membrane immune receptors Cf4 and Cf9 (Rowland et al., 2005). TRK1b (tomato protein kinase 1b) is involved in plant resistance to necrotrophic fungi via ethylene signaling pathway (Abuqamar et al., 2008). Furthermore, TRK1 (TRK1b-related kinase 1) regulates plant immunity by directly acting downstream of cell surface receptors SlLYK1 (LysM receptor like kinase 1) and SlCLV1 (CLAVATA1, Jaiswal et al., 2022). *CaPIK1* gene from RLCK VII subfamily in pepper is homologous to the Arabidopsis genes *AtPBL5*/*6*. Its high expression in pepper promotes the accumulation of ROS and enhances plant immune response against *Xanthomonas campestris*. Conversely, silencing this gene reduces plant resistance to pathogens (Kim and Hwang, 2011). In wheat, *TaRLCK1B* gene from TaRLCK VII subfamily shows significantly higher expression in varieties resistant to sharp eyespot compared to susceptible ones. Silencing this gene reduces plant resistance to *Rhizoctonia cerealis* and leads to increased H_2_O_2_ level (Wu et al., 2020). In cassava, *MeBIK1* gene from RLCK VII subfamily is expressed at low level under normal conditions, but is strongly induced by flagellin flg22. *MeBIK1* can restore the weakened pattern triggered immunity (PTI) response of *atbik1* mutants, and its overexpression enhances resistance to *Xanthomonas axonopodis* in *A. thaliana* (Li et al., 2018).

Broad beans (*Vicia faba* L.), members of the Leguminosae family, are rich in proteins, amino acids, vitamins, and mineral elements. They have a strong ability to fix nitrogen and serve as important crops for food, vegetables, animal feed, and green manure. China accounts for about 34% of the global cultivation area of broad beans and leads the world in production, contributing approximately 36% of the global yield (http://www.fao.org). Fungal diseases are major constraints on broad bean yield and quality, with common diseases including chocolate spot, root rot, rust, etc. In recent years, leaf spot and downy mildew have emerged as new diseases in broad beans, becoming increasingly severe and causing significant economic losses. Although RLCK VII subfamily has been extensively studied in model plants like Arabidopsis and rice, research on its family members, molecular characteristics, and biological functions in broad beans is still lacking. Thus, we can draw on the theoretical framework established in Arabidopsis and employ mature bioinformatics and transgenic techniques to conduct research. This study identified and characterized the VfRLCK VII subfamily across the broad bean genome, analyzed their structural and regulatory properties, assessed their expression in response to *Alternaria alternata* infection, and validated the disease-resistance role of *VfRLCK VII4*. These findings lay a foundation for molecular breeding of disease-resistant cultivars.

## 2 Materials and methods

### 2.1 Identification of RLCK VII subfamily genes in broad beans

The complete genome data of broad beans (*Vicia faba* genome v1.0, released on May 2023) was downloaded from the broad bean genome database (https://projects.au.dk/fabagenome/genomics-data) , and 46 RLCK VII protein sequences of Arabidopsis were obtained from the TAIR database (https://www.arabidopsis.org). ‘Blast Several Sequences to a Big File’ function in TBtools software (e-value ≤ 1e^-10^, identity>50%) was utilized to identify protein sequences containing conserved kinase domains of RLCK VII (Pfam ID: PF00069). The identified protein sequences were then submitted to Conserved Domain Database (CDD) of NCBI (https://www.ncbi.nlm.nih.gov/cdd) and Pfam database (https://www.ebi.ac.uk/interpro/entry/pfam/#table) for conserved domain analysis. Subsequently, SMART database (http://smart.embl.de/) was used for excluding sequences with different structural features. Through the above process, the identification of RLCK VII subfamily genes in broad bean was completed (Cen et al., 2023).

### 2.2 Physical and chemical properties and subcellular localization analysis of VfRLCK VII proteins

Expasy ProtParam (https://web.expasy.org/protparam/) (Wilkins et al., 1999) was employed to analyze physicochemical properties of RLCK VII proteins in broad beans, mainly including number of amino acid, molecular weight, isoelectric point (pI), instability index, and aliphatic index. Meanwhile, subcellular localization of RLCK VII proteins was predicted using Cell-PLoc 2.0 (http://www.csbio.sjtu.edu.cn/bioinf/Cell-PLoc-2/).

### 2.3 Phylogenetic analysis of VfRLCK VII proteins

To explore the relationship between RLCK VII members in broad beans and Arabidopsis, multiple sequence alignment of their protein sequences was conducted using Clustal X software (Thompson et al., 1997). A phylogenetic tree was then constructed with MEGA 11.0 software (Kumar et al., 2018) based on Neighbor-Joining (NJ) method, after removing low-quality regions, applying P-distance model, and setting Bootstrap set to 1000. In addition, the phylogenetic tree was visualized and annotated using Evolview online software (http://www.evolgenius.info/evolview) (Subramanian et al., 2019), which also facilitated the classification of VfRLCK VII proteins.

### 2.4 Gene structure, conserved domains, and conserved motifs analysis

To analyze gene structure and clarify the positions of exons and introns of *VfRLCK VII* genes, TBtools software was exploited. And protein domains of VfRLCK VIIs were confirmed through CDD database of NCBI. Concurrently, conserved motifs of VfRLCK VIIs were identified by online software MEME (https://meme-suite.org/meme/tools/meme) (Bailey et al., 2009), and the maximum motif was set to 10. Finally, the above results were visualized using the ‘Gene Structure View’ function of TBtools software (Chen et al., 2020).

### 2.5 Chromosome distribution, gene duplication, and collinearity analysis

Using TBtools software, a chromosome localization map of VfRLCK VII subfamily genes was generated. MCScanX function of TBtools was applied to detect the tandem and segmental duplication events and to perform collinearity analysis of *VfRLCK VII* genes in the broad bean genome. Tandem duplications were defined as two or more homologous genes located within a 100 kb region on the same chromosome. A collinearity map was created using the TBtools Circos function. In addition, non-synonymous substitution rate (*Ka*), synonymous substitution rate (*Ks*), and *Ka*/*Ks* ratios were calculated using TBtools Simple Ka/Ks Calculator (default parameters) to assess selection pressure. Genome data of *Medicago truncatula* (GenBank: GCA_003473485.2) and *Pisum sativum* (GCF_024323335.1) were downloaded from NCBI (https://www.ncbi.nlm.nih.gov/datasets/genome/) , and collinearity analysis among the three legume plants was conducted using MCScanX of TBtools.

### 2.6 Cis-acting elements analysis

In order to identify the cis-acting elements contained in the promoter regions of *VfRLCK VII* genes, 2000 bp DNA sequences upstream of the initiation codon of CDS were extracted by TBtools software. Then the sequences were submitted to PlantCARE (http://bioinformatics.psb.ugent.be/webtools/plantcare/html/) (Lescot et al., 2002) to predict promoter cis-acting elements. Finally, the results were visualized using TBtools.

### 2.7 Expression characteristics analysis

Broad bean germplasm CD-006 and *A. alternata* strain VFL10, preserved in Jiangsu Yanjiang Institute of Agricultural Sciences, were used as experimental materials. The 5-day cultured fungal cakes (diameter of 9mm) of VFL10 were taken from potato dextrose agar (PDA) medium and inoculated onto the fully expanded living broad bean leaves, each having four leaves and one heart leaf (3 weeks post-germination). The inoculated plants were kept in darkness at 25°C, with humidity maintained by water spraying and a humidifier. Then the leaves at 0, 6, 12 and 24 h after inoculation were sampled and sent to Guangzhou Jidiao Technology Service Co., Ltd. for transcriptome sequencing, the leaves collected at 0 h were used as the control. Each sample was repeated three times. RNA was extracted using Trizol reagent (Invitrogen, Carlsbad, CA, USA), libraries were constructed using Poly (A) capture method, and sequencing was performed on an Illumina Novaseq6000 platform (paired-end 250 bp). The raw transcriptome data were uploaded to the NCBI SRA database with accession number PRJNA1327605. Expression levels of each transcript were quantified using FPKM (fragments per kill base per million mapped reads) values, which were then used to generate heatmaps showing expression patterns of *VfRLCK VII* genes. Differential expression analysis was performed, genes with FDR (false discovery rate)<0.05 and |log_2_FC (fold change)| > 1 were considered as differentially expressed genes (DEGs). Eight genes of them were selected for quantitative verification. Primer sequences for qRT-PCR were listed in **Supplementary Table S1**. Each sample was subjected to three biological replicates.

### 2.8 Validation of disease resistance function of *VfRLCK VII4* gene

Based on genome identification and transcriptome sequencing results, *VfRLCK VII4* gene was selected for function validation related to disease resistance. The ORF (open reading frame) of *VfRLCK VII4* gene was cloned and inserted into the pCAMBIA-GFP vector (obtained from Shanxi AUG Biotechnology Co., Ltd.) via homologous recombination. The empty vector was digested with *SacI* and *XbaI* restriction enzymes to construct genetic transformation vector pCAMBIA-*VfRLCK VII4*. Primer sequences for gene cloning and vector construction were listed in **Supplementary Table S1**.

Before genetic transformation of tobacco, the expression vector was firstly transformed into *Agrobacterium tumefaciens* strain *GV3101*. Successfully transformed strains were used to infect tobacco cultivar SR1 through tissue culture method. SR1 seeds were sterilized with 75% ethanol and NaClO solution to obtain sterile seedlings. Following Agrobacterium infection of explants, co-cultivation, bud induction, differentiation, and rooting steps were performed to regenerate complete plants. Once small plants generated, genomic DNA was extracted from them using Simgen Plant DNA Kit (Hangzhou Simgen Biotechnology Co., Ltd.), and positive transformants were detected using gene specific primers, resulting in the T0 generation. Homozygous T3 lines were confirmed by PCR genotyping and gene-specific qRT-PCR analysis. Three lines with high *VfRLCK VII4* expression were selected for further disease resistance testing.

To assess disease resistance, injury-induced inoculation method was used to infect tobacco plants with *A. alternata*. When plants reached the 7–8 leaf stage, healthy leaves without yellowing at the base were selected. After surface sterilization with alcohol and rinsing with sterile water, leaves were pricked and inoculated with fungal cakes, then cultivated in darkness at 25°C. At 5 d after inoculation, disease incidence of leaves was observed, lesion diameters were measured using the cross method manually, and lesion areas were calculated (Daley et al., 2017). Each tobacco line included five biological replicates, and data were analyzed using a two-tailed Student’s *t*-test.

## 3 Results

### 3.1 Identification of RLCK VII subfamily members in *V. faba*

Using bioinformatics method, 45 genes encoding VfRLCK VII proteins were identified in the broad bean genome. These genes were named as *VfRLCK VII1*-*VfRLCK VII45* based on their chromosomal locations. Analysis of physicochemical properties of VfRLCK VII proteins revealed that their amino acid sequences ranged from 296 to 595 aa in length, with molecular weight between 32530.79 and 65018.03 Da. Their theoretical isoelectric point (pI) varied from 4.63 to 9.78, instability index ranged from 23.23 to 46.89, and fat index ranged from 56.27 to 96.45. Subcellular localization predictions indicated that 39 proteins were located in the nucleus, and 6 proteins were located in chloroplasts (**Supplementary Table S2**).

### 3.2 Phylogenetic analysis of RLCK VII subfamily members in *V. faba* and *A. thaliana*

**Figure 1:**
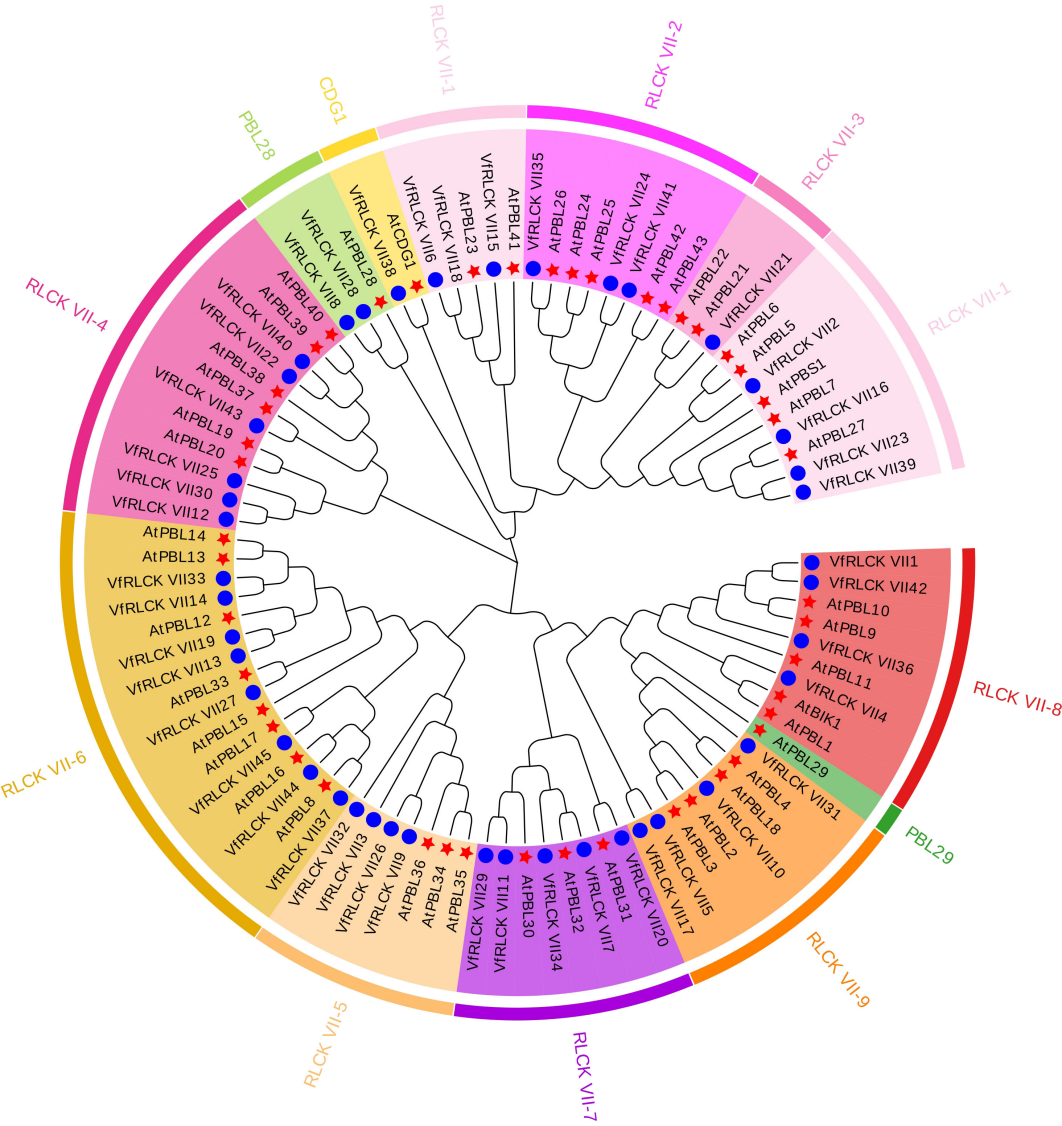
Phylogenetic analysis of RLCK VII subfamily members in *V. faba* and *A. thaliana*. Different background colors represented different subgroups. The inner circle represented the names of RLCK VII proteins, and the outer circle represented different subgroup classifications. The blue circles represented VfRLCK VII proteins, and the red asterisks represented AtRLCK VII proteins.

In order to reveal the evolutionary relationships among RLCK VII subfamily members in broad beans, a phylogenetic tree was constructed using 45 VfRLCK VII protein sequences alongside with 46 AtRLCK VII protein sequences from Arabidopsis. According to the classification system of AtRLCK VII proteins, VfRLCK VII proteins were divided into 9 subgroups (VfRLCK VII-1 - VfRLCK VII-9) and 3 members (VfRLCK VII8, VfRLCK VII28, and VfRLCK VII38) (**Figure 1**). The majority of VfRLCK VII proteins were clustered in subgroups RLCK VII-6 (8), RLCK VII-1 (7), and RLCK VII-4 (6) subgroups, followed by RLCK VII-7 (5), RLCK VII-5 (4), RLCK VII-8 (4), and RLCK VII-9 (4) subgroups. Fewer proteins were found in RLCK VII-2 (3) and RLCK VII-3 (1) subgroups. Moreover, two proteins VfRLCK VII8 and VfRLCK VII28 were clustered with AtPBL28, while protein VfRLCK VII38 was clustered with AtCDG1, but no protein was clustered with AtPBL29.

### 3.3 Gene structure, conserved domains, and motif analysis of RLCK VII subfamily members in *V. faba*

**Figure 2:**
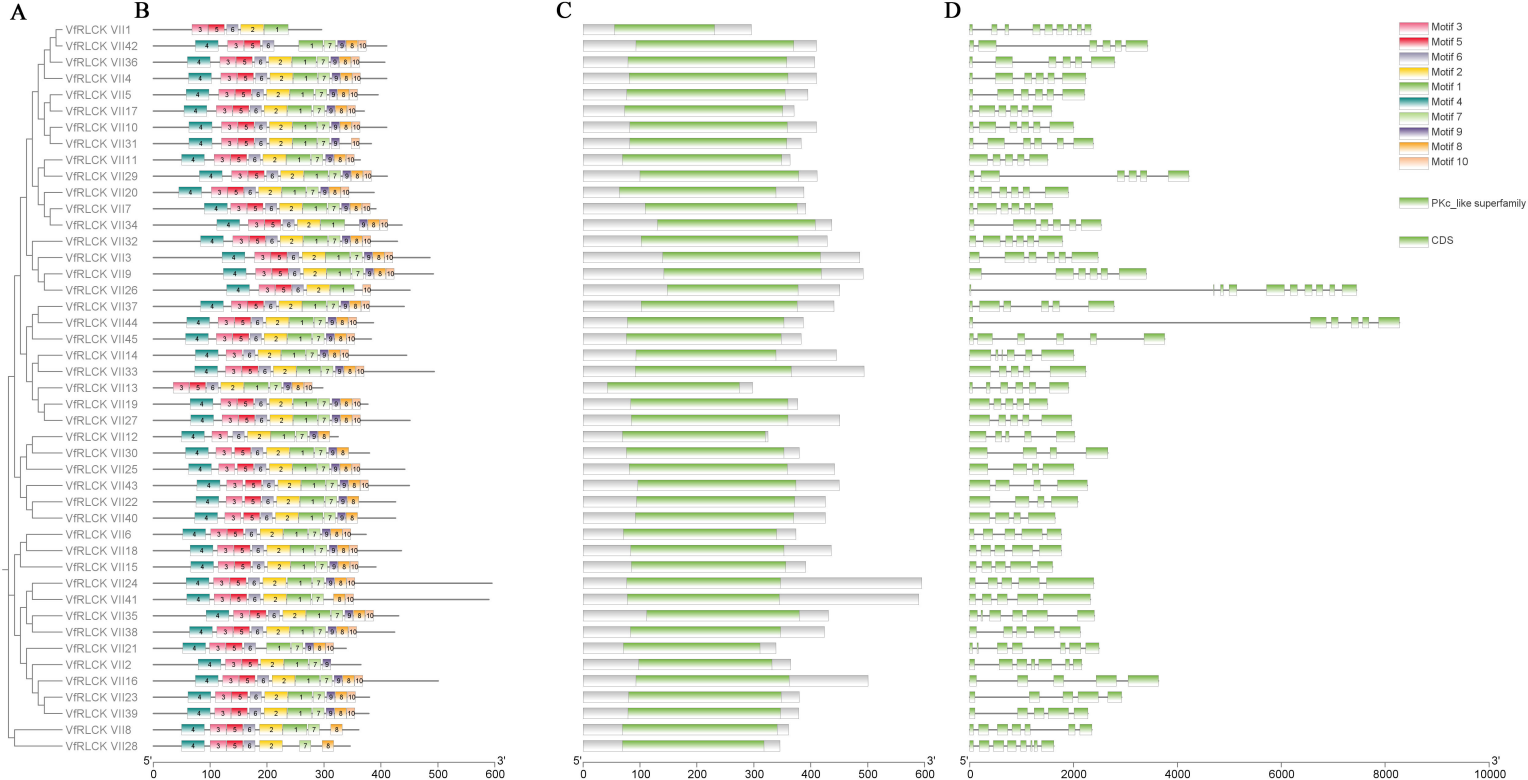
Gene structure, conserved domains, and motif analysis of VfRLCK VII subfamily members. **(A)** Phylogenetic tree of VfRLCK VII subfamily members; **(B)** Conserved motifs of VfRLCK VII subfamily members; **(C)** Conserved domains of VfRLCK VII subfamily members; **(D)** Gene structure of VfRLCK VII subfamily genes.

A phylogenetic tree was constructed for 45 VfRLCK VII proteins (**Figure 2A**) and their conserved motifs were visualized. The results showed that motif3 was present in all VfRLCK VII proteins, motif1 and motif6 appeared in 44 proteins, motif2, motif4, and motif5 were found in 43 proteins, motif7 in 42 proteins, motif8 in 41 proteins, motif9 in 40 proteins, and motif10 in 37 proteins. Most VfRLCK VII proteins (86.67%) contained 9–10 motifs, a smaller portion (11.11%) had 7–8 motifs, and only one protein, VfRLCK VII1, contained 5 motifs (**Figure 2B**).

Conserved domain analysis of 45 VfRLCK VII proteins showed that they were highly conserved, each containing a single PKc_like superfamily domain (**Figure 2C**).

In addition, gene structure analysis based on the broad bean genome sequence revealed that *VfRLCK VII* genes contained 4–10 exons. Most genes (75.56%) had 5–6 exons, 5 genes (11.11%) had 4 exons, 3 genes *VfRLCK VII21*, *VfRLCK VII2*, and *VfRLCK VII8* had 7 exons, one gene *VfRLCK VII28* had 8 exons, one gene *VfRLCK VII1* had 9 exons, and one gene *VfRLCK VII26* had 10 exons (**Figure 2D**).

### 3.4 Chromosome localization and collinearity analysis of RLCK VII subfamily members in *V. faba*

**Figure 3:**
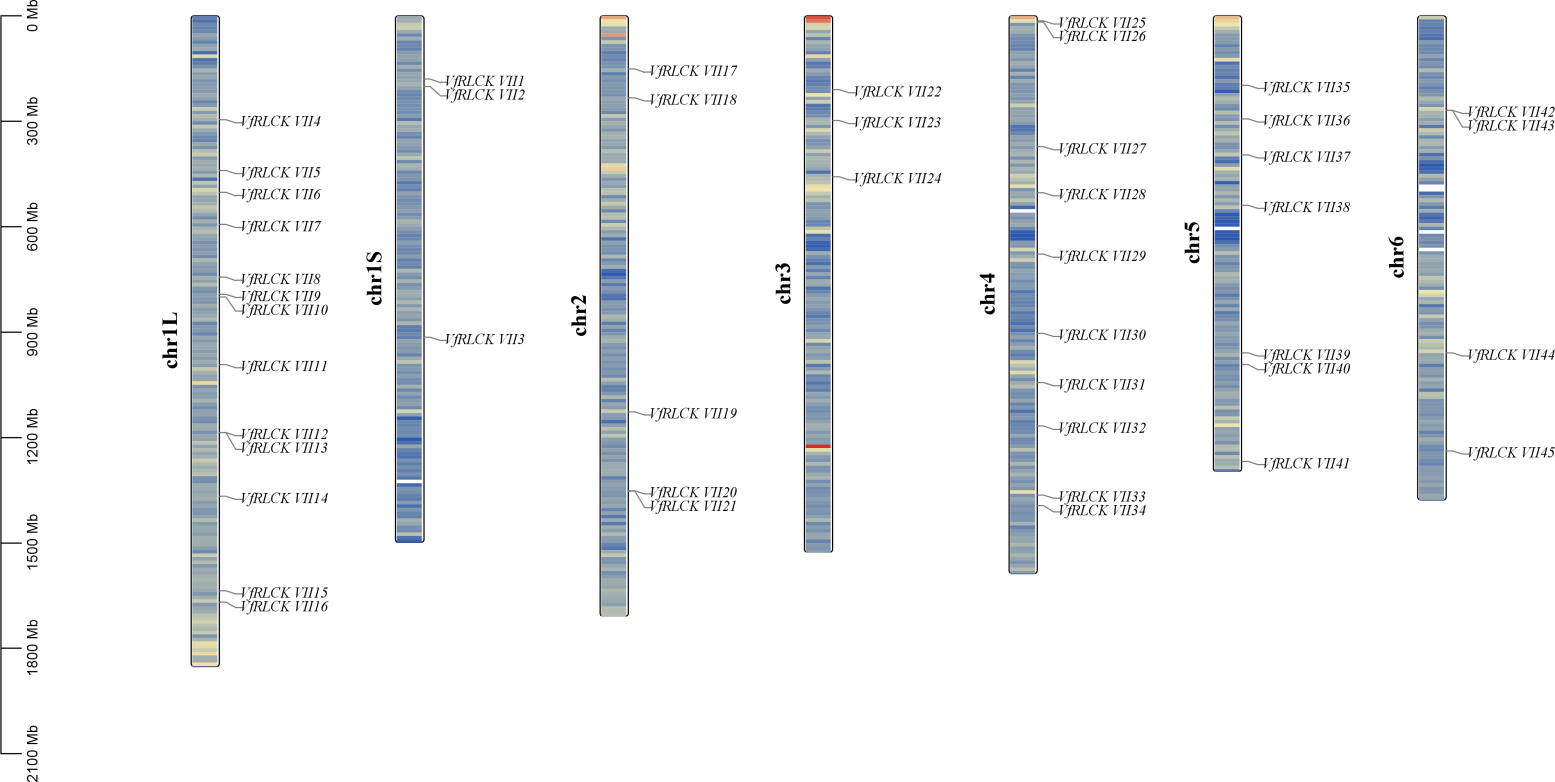
Chromosome distribution of RLCK VII subfamily genes in broad beans.

A chromosomal map of RLCK VII subfamily genes in broad beans was created by TBtools, revealing that 45 genes were unevenly distributed on 6 chromosomes (**Figure 3**). There were 16 genes *VfRLCK VII1* - *VfRLCK VII16* on chromosome 1, 5 genes *VfRLCK VII17* - *VfRLCK VII21* on chromosome 2, 3 genes *VfRLCK VII22* - *VfRLCK VII24* on chromosome 3, 10 genes *VfRLCK VII25* - *VfRLCK VII34* on chromosome 4, 7 genes *VfRLCK VII35* - *VfRLCK VII41* on chromosome 5, and 4 genes *VfRLCK VII42* - *VfRLCK VII45* on chromosome 6.

**Figure 4:**
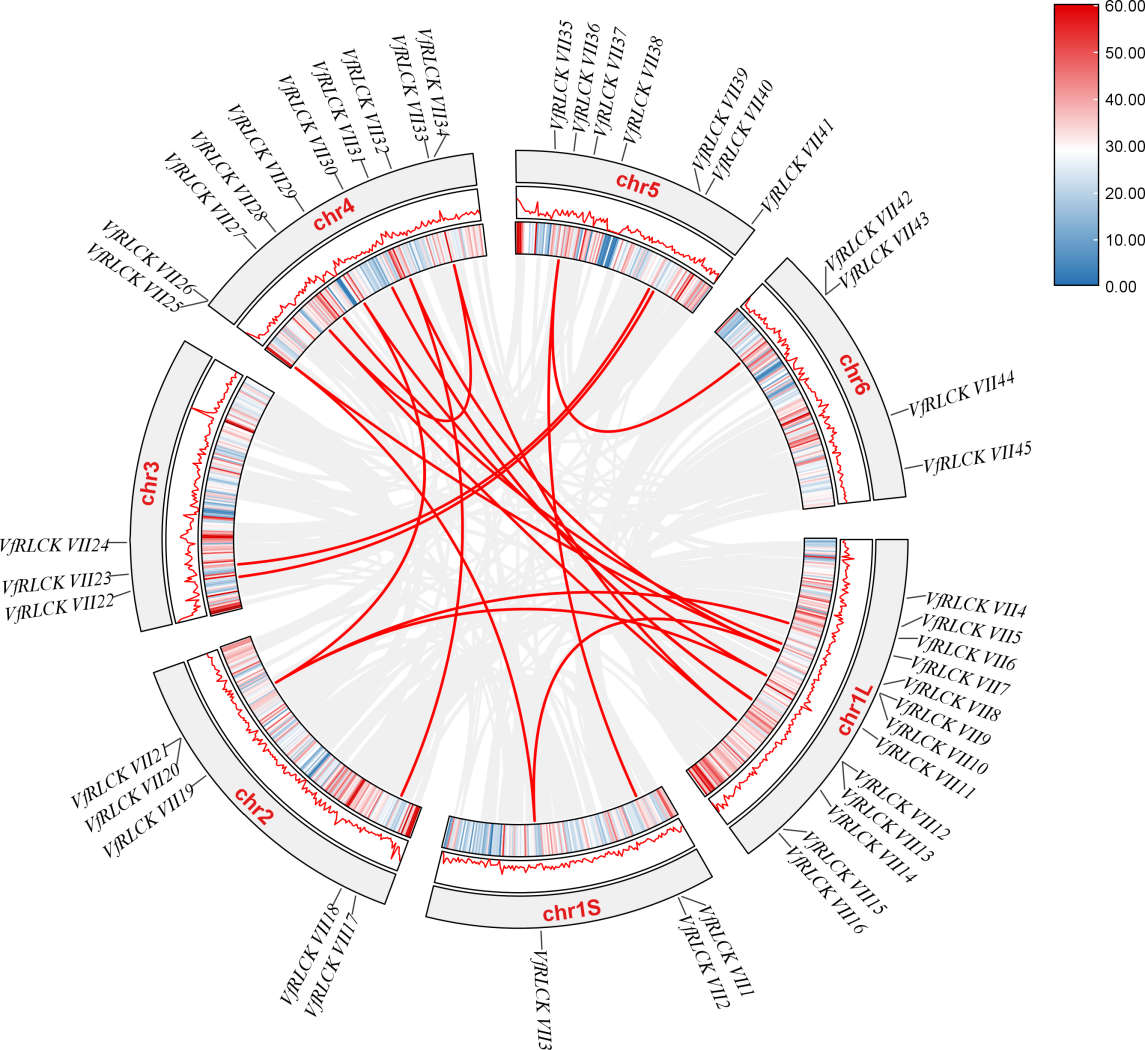
Collinearity relationship of RLCK VII subfamily genes in broad beans.

For further exploration of the evolutionary relationship among RLCK VII subfamily genes in broad beans, TBtools was used to analyze duplication events of 45 *VfRLCK VII* genes. The analysis identified 18 segmental duplication events across different chromosomes (**Figure 4**), but no tandem duplication event was detected (**Figure 3**), suggesting that segmental duplication contributed to the expansion of RLCK VII subfamily. Additionally, to assess environmental selection pressure after gene duplication, *Ka*/*Ks* values of 18 duplicated gene pairs were calculated (**Supplementary Table S3**). As a result, except for one duplicated gene pair with a *Ka*/*Ks* value of NaN, the *Ka*/*Ks* values of all other duplicated gene pairs were less than 1, indicating that these 17 duplicated gene pairs underwent purification selection during the evolutionary process.

**Figure 5:**
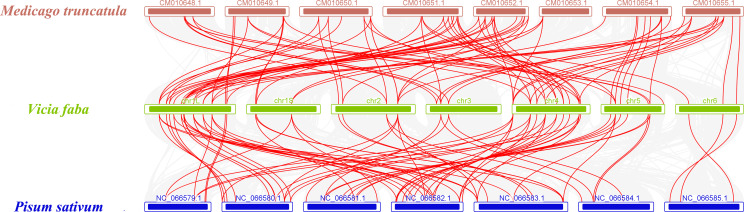
Collinearity relationship of RLCK VII subfamily genes in *V. faba*, *M. truncatula*, and *P. sativum*.

To gain deeper insight into the evolutionary mechanism of RLCK VII subfamily genes, collinearity analysis was performed on this subfamily genes of *Vicia faba*, *Medicago truncatula* and *Pisum sativum* (**Figure 5**). The results showed 81 homologous RLCK VII gene pairs between *V. faba* and *M. truncatula*, and 72 homologous pairs between *V. faba* and *P. sativum*, suggesting a close evolutionary relationship among these species.

### 3.5 Analysis of cis-acting elements in the promoters of VfRLCK VII subfamily genes

**Figure 6:**
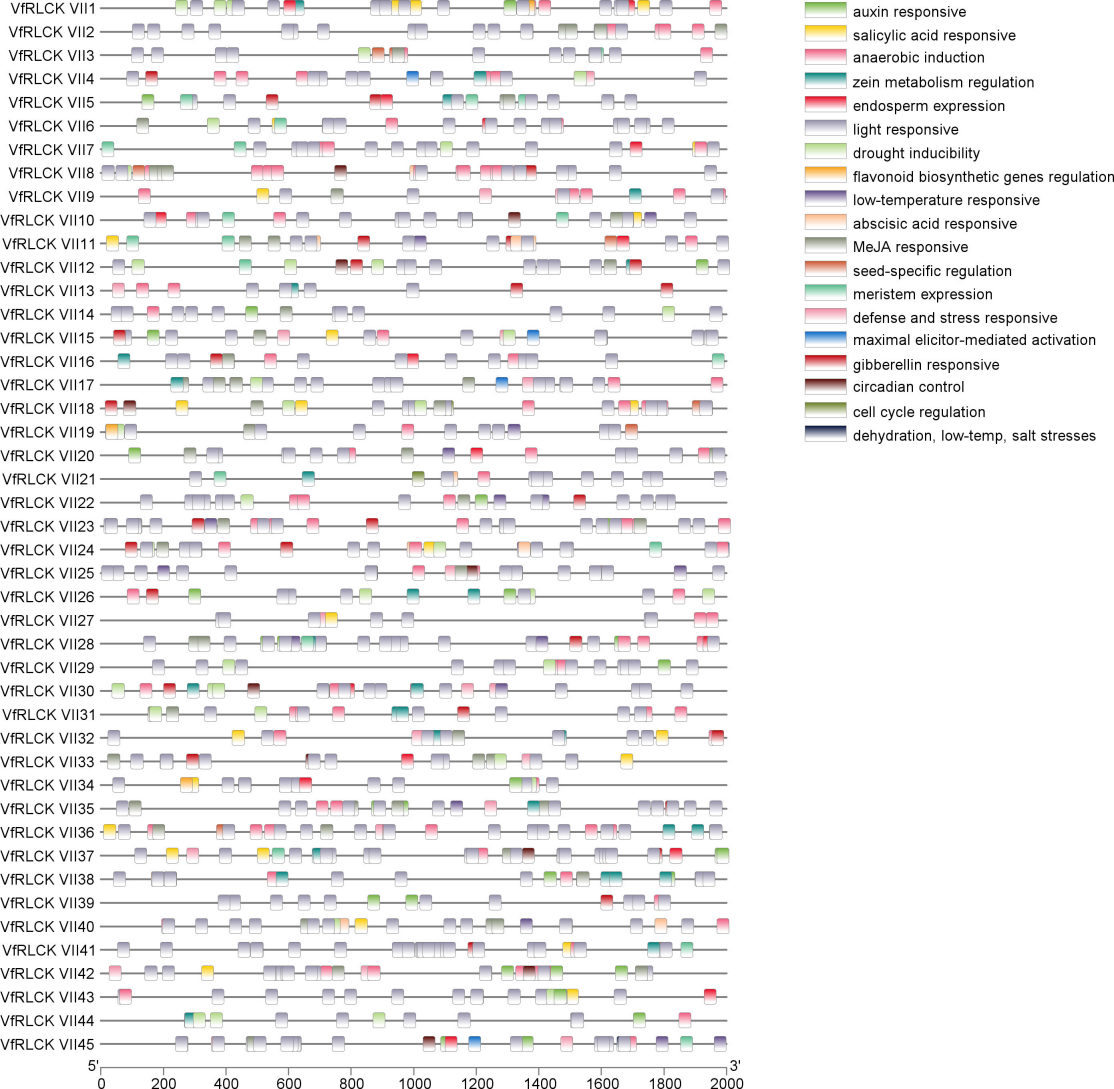
Analysis of cis-acting elements of RLCK VII subfamily genes in broad beans.

Analysis of the 2000 bp DNA sequences upstream of *VfRLCK VII* genes revealed that most promoters did not overlap with neighboring genes. However, promoters of *VfRLCK VII2* and *VfRLCK VII43* overlapped with neighboring genes, with 1539 and 1114 bp overlap, respectively. The identified cis-acting elements could be classified into 4 categories: light responsive elements, plant hormone responsive elements, stress responsive elements, and growth and development regulatory elements (**Figure 6**). All *VfRLCK VII* genes contained light responsive elements, indicating that their expression was generally regulated by light. Regarding plant hormone responsive elements, 22 *VfRLCK VII* genes contained auxin responsive elements, 22 *VfRLCK VII* genes contained gibberellin responsive elements, 19 *VfRLCK VII* genes contained salicylic acid responsive elements, 33 *VfRLCK VII* genes contained abscisic acid responsive elements, and 33 *VfRLCK VII* genes contained MeJA responsive elements, suggesting that the expression of *VfRLCK VII* genes might be influenced by hormone content in plants. In terms of stress responsive elements, 17 *VfRLCK VII* genes contained defense and stress responsive elements, 42 *VfRLCK VII* genes contained anaerobic induction regulatory elements, 23 *VfRLCK VII* genes contained drought inducibility regulatory elements, 15 *VfRLCK VII* genes contained low-temperature responsive elements, and 1 *VfRLCK VII* gene contained dehydration, low-temperature, and salt stresses responsive elements, implying that *VfRLCK VII* genes might be involved in various stress resistance processes in broad beans. Furthermore, 37 *VfRLCK VII* genes contained growth and development regulatory elements, indicating that most *VfRLCK VII* genes were involved in the growth and development of broad beans. In addition, except for light responsive elements, anaerobic induction regulatory elements, abscisic acid responsive elements and MeJA responsive elements were remarkably rich in some *VfRLCK VIIs* genes (**Supplementary Table S4**).

### 3.6 Expression characteristics analysis of *VfRLCK VII* genes in response to *A. alternata* infection

**Figure 7:**
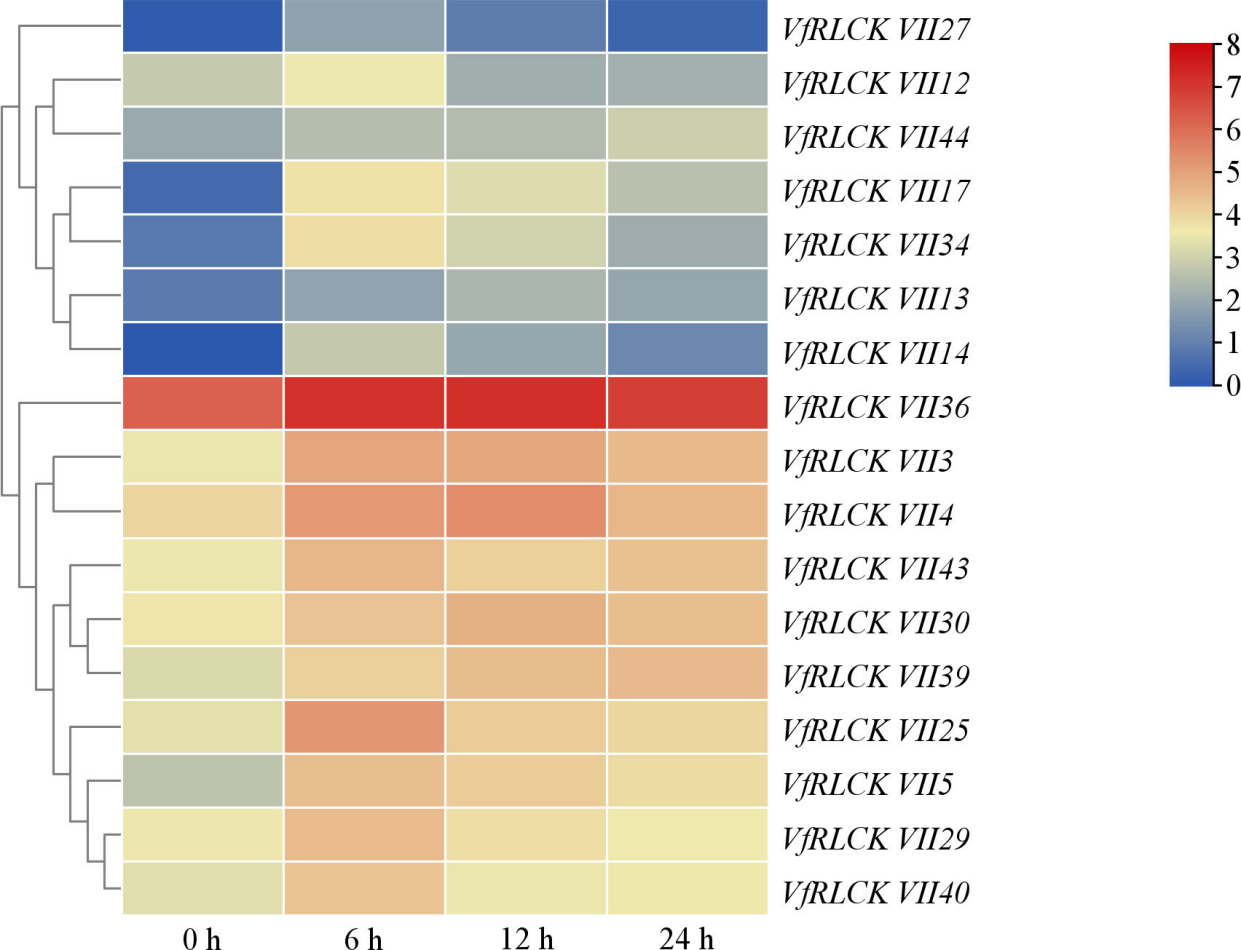
17 *VfRLCK VII* genes were up-regulated after infection with *A. alternata*. The color bar in the upper right corner represented FPKM values based on log2 normalization, while blue to red indicated gene relative expression levels from low to high. *p*_adj_<0.05.

**Figure 8:**
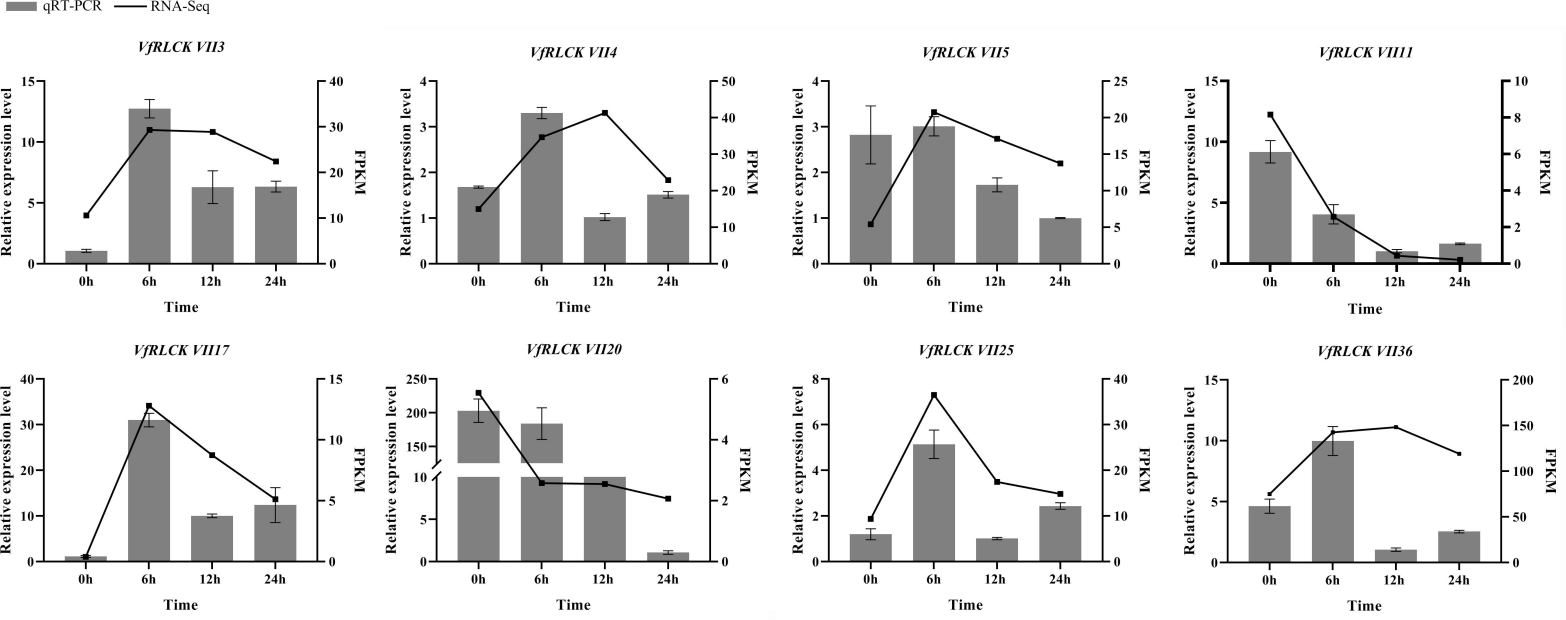
qRT-PCR analysis of *VfRLCK VII* genes in response to infection with *A. alternata* in broad beans.

In order to investigate the response of *VfRLCK VII* genes to pathogens, their expression patterns under infection with *A. alternata* were analyzed (**Supplementary Figure S1**). Within 45 genes, 21 genes showed differential expression. Among them, 17 genes were initially up-regulated and then down-regulated after infection (**Figure 7**), potentially playing a positive role in disease resistance of broad beans. Conversely, 4 genes *VfRLCK VII11*, *VfRLCK VII20*, *VfRLCK VII28*, and *VfRLCK VII42* were down-regulated after infection, possibly acting as negative regulators of broad bean disease resistance. Eight genes were randomly selected for qRT-PCR validation, and the quantitative results were consistent with the transcriptome sequencing results (**Figure 8**).

### 3.7 Analysis of disease resistance function of *VfRLCK VII4*

**Figure 9:**
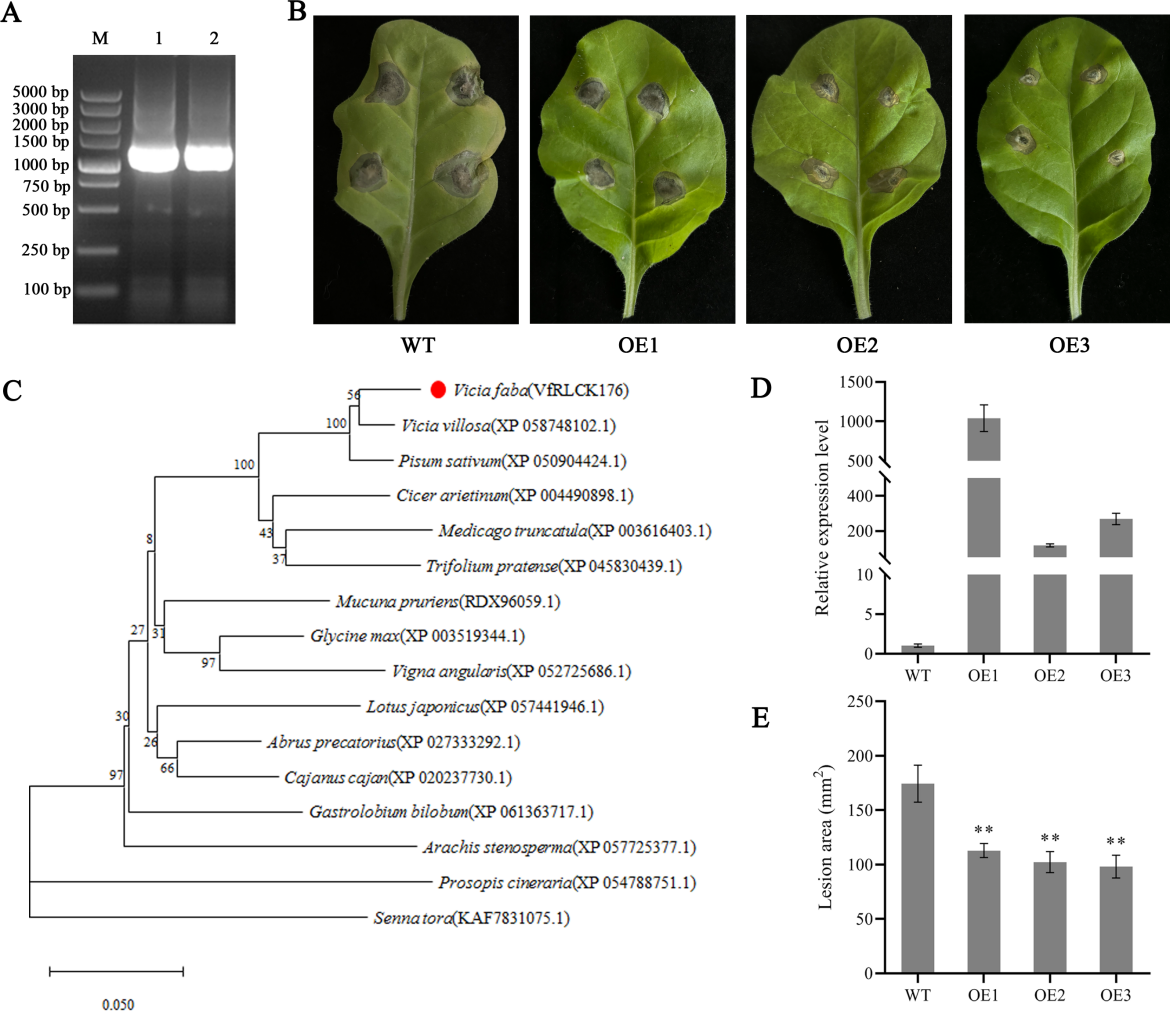
*VfRLCK VII4* gene enhanced tobacco resistance to *A. alternata*. **(A)** Cloning of *VfRLCK VII4* gene; M represented 5000 bp DNA marker, 1 and 2 represented PCR amplified product of *VfRLCK VII4* gene; **(B)** Lesion phenotype at 5 d after infection; **(C)** Phylogenetic tree of VfRLCK VII4 (VfRLCK176) protein; **(D)** Relative expression level of *VfRLCK VII4* in tobacco; **(E)** Lesion areas at 5 d after infection. ** indicated significant difference at the *p* < 0.01 level.

Both transcriptome and quantitative results showed that *VfRLCK VII4* gene was up-regulated at 6 h after infection with *A. alternata* before being down-regulated. Phylogenetic analysis of RLCK VII subfamily members placed VfRLCK VII4 within the RLCK VII-8 subgroup, showing close homology to AtBIK1 and AtPBL1 from Arabidopsis. The two proteins are involved in immune response in Arabidopsis, and AtBIK1 is the most extensively studied, it is a central component of PTI and integrates multiple PRR signals. Therefore, *VfRLCK VII4* gene was chosen to validate its role in disease resistance in broad beans. The ORF of *VfRLCK VII4* gene was 1233 bp, encoding 411 amino acids (**Figure 9A**). The phylogenetic tree showed that VfRLCK VII4 was most closely related to RLCK176 in *V. villosa*, suggesting it could also be named as VfRLCK176 (**Figure 9C**). Three transgenic tobacco lines with high *VfRLCK VII4* expression levels were selected for disease resistance identification (**Figure 9D**). The results indicated that after inoculation with *A. alternata*, wild type leaves showed faded green areas and water soaked lesions, whereas the symptoms of transgenic lines were milder (**Figure 9B**). Lesion area of wild-type plants was 174.32 mm², while lesion areas of transgenic lines OE1, OE2, and OE3 were significantly smaller at 112.90 mm², 102.29 mm², and 98.13 mm², respectively (**Figure 9E**). These results indicated that VfRLCK VII4 could positively regulate tobacco resistance to *A. alternata*.

## 4 Discussion

Members of RLCK VII subfamily play crucial roles in plant kinase mediated signal transduction and various cellular processes, including plant growth and development, as well as defense against biotic and abiotic stresses, particularly in immune responses triggered by pathogen associated molecular patterns (PAMPs). This study identified 45 members of RLCK VII subfamily in the broad bean genome, which was fewer than 46 in Arabidopsis (Lin et al., 2015) and 72 in cotton (Liu et al., 2023). Despite the broad bean genome being approximately 13 Gb, larger than those of Arabidopsis and cotton, this discrepancy suggests that gene family size is more influenced by species-specific gene duplication and loss events than by overall genome size. Despite the large genome size of broad beans, the species is diploid and exhibits an extremely loose genomic structure characterized by a high abundance of repetitive sequences interspersed between genes, resulting in extensive intergenic distances. This genomic organization may account for the relatively small gene family size in broad beans.

Utilizing the classification framework established for Arabidopsis RLCK VII members, VfRLCK VII proteins in broad beans were similarly divided into 9 subgroups. The number of members in each subgroup was similar to that of Arabidopsis, which might be related to the similar number of RLCK VII members between the two species. Phylogenetic analysis of RLCK VII members in broad beans and Arabidopsis suggested that RLCK VII proteins with high sequence similarity clustering within the same clade were likely to share analogous biological functions. For example, VfRLCK VII proteins clustering with Arabidopsis homologs (e.g., PBL27, PBS1, BIK1) likely performed similar roles in immune responses (Li et al., 2021; Liu et al., 2019; Ma et al., 2020; Pottinger et al., 2020; Sreekanta et al., 2015). Besides, potential functional redundancy might exist among genes in the same subgroup (**Supplementary Table S5**). The high conservation of motifs, domains, and exon structures among VfRLCK VII members suggested strong evolutionary constraints, reflecting critical roles in immune signaling. Notably, motif conservation might relate to protein-protein interactions in signaling pathways.

Chromosome segmental duplication and tandem duplication provide original genetic materials for the generation of new genes and are two important factors in gene family expansion, which facilitate phenotypic changes and promote species evolution (Freeling, 2009; Wang et al., 2019). Collinearity analysis conducted in this study indicated that segmental duplications were the main driver of VfRLCK VII family expansion, while tandem duplications were absent. The new genes generated by gene duplication may become inactive pseudogenes, may work together with existing genes, or may produce new functions, thereby promoting functional diversification and adaptability to diverse stresses. And the *Ka*/*Ks* values of 18 duplicated gene pairs were calculated, all duplicated gene pairs exhibited *Ka*/*Ks* ratios <1, indicating strong purifying selection and functional conservation (**Supplementary Table S3**, Cao and Shi, 2012). This finding supports the idea that *RLCK VII* genes maintain essential roles in plant immunity.

Promoter analysis of *VfRLCK VII* genes identified four major types of cis-acting regulatory elements: light responsive, hormone responsive, stress responsive, and growth/development regulatory elements. Hormone mediated signal transduction plays an important part in plant resistance to pathogen invasion, among which salicylic acid, jasmonic acid, and ethylene are three main hormones used by plants to combat pathogens. Salicylic acid is typically involved in the defense response of plants against biotrophic and hemi-biotrophic pathogens, establishing systemic acquired resistance (SAR), while jasmonic acid is typically involved in the defense response against necrotrophic and hemi-biotrophic pathogens (Betsuyaku et al., 2018). Among 45 *VfRLCK VII* genes, MeJA responsive elements were remarkably rich, present in 33 genes, suggesting their involvement in hormone-mediated immune responses. Moreover, 17 *VfRLCK VII* genes contained defense and stress responsive elements, they might also be involved in the defense response of pathogens. Interactions between hormone responsive elements and stress responsive elements may synergistically enhance transcription activation of defense genes, or alternatively exert mutual repression, thereby enabling plants to finely tune their responses to a variety of biotic and abiotic stresses.

Seventeen *VfRLCK VII* genes were up-regulated, while four were down-regulated after *A. alternata* infection, with twelve genes peaking at 6 h post-inoculation. Notably, *VfRLCK VII4*, *VfRLCK VII14* and *VfRLCK VII40*, homologous to key Arabidopsis immune regulators *AtBIK1*, *AtPBL1*, *AtPBL13–14* and *AtPBL39*-*40* (Kong et al., 2016; Lal et al., 2018; Li et al., 2021; Lin et al., 2015), represented prime candidates for disease resistance function studies.

RLCK176 plays a central role in plant immunity, especially in monocotyledonous plants such as rice and wheat. Its functions include integrating multiple immune signaling pathways, positively regulating defense responses, and balancing growth and immunity. In rice, OsRLCK176 interacted with the chitin receptor OsCERK1 to regulate the immune responses triggered by chitin and peptidoglycan, including ROS bursts and defense gene expression (Ao et al., 2014). RLCK176, alongside with other RLCKs, could participate in regulating the PTI and ROS burst responses induced by chitin and flagellin flg22 (Li et al., 2017; Zhou et al., 2016). In addition, OsRLCK176 promoted ROS production by phosphorylating the NADPH oxidase OsRbohB, thereby enhancing the killing efficacy against pathogen. OsRLCK176 could also form complex with SDS2 and SPL11, regulating cell death and immune response (Fan et al., 2018). Recent study has identified OsCPK17 and OsPUB12 as positive and negative immune regulatory factors, respectively, that fine tune defense responses by maintaining the homeostasis of OsRLCK176 in rice (Mou et al., 2024). In wheat, TaRLCK176 interacted with sulfite oxidase TaSO-7D to positively regulate wheat resistance to stripe rust (Qin, 2024). In our study, VfRLCK VII4 (VfRLCK176) conferred enhanced resistance to *A. alternata* in transgenic tobacco, consistent with its homology to BIK1/PBL1. Interestingly, among 3 transgenic lines, OE1 line showed the highest gene expression but the lowest resistance phenotype (**Figures 9B, D, E**). This observation was contrary to the conventional expectation of a direct correlation between gene expression and functional gain, indicating that the function of VfRLCK176 was not governed by simple linear regulation, but was subjected to homeostasis balance control. Further studies, including protein-protein interaction assays and ROS quantification, are needed to elucidate the underlying molecular mechanism. Cis-element analysis of the *VfRLCK176* promoter revealed defense and stress responsive motifs, supporting its role in immune regulation, this hypothesis could be further validated through promoter activity assays.

## 5 Conclusion

Broad beans are a crop with both nutritional and economic value, however, their production is severely affected by fungal diseases. Members of the RLCK VII subfamily play a crucial part in plant defense response. In this study, members of RLCK VII subfamily in broad beans were identified, and their physicochemical properties, evolutionary relationships, gene structure, chromosome distribution, cis-acting elements, and expression characteristics were analyzed. It was found that there were a total of 45 members in the broad bean RLCK VII subfamily, of which 21 members were differentially expressed after infection by *A. alternata*. Among the above 21 members, *VfRLCK VII4* (*VfRLCK176*) was up-regulated at 6 h after pathogen infection, and it was closely homologous to *AtBIK1* and *AtPBL1* in Arabidopsis, as well as *OsRLCK176* in rice. Heterologous transformation of *VfRLCK VII4* in tobacco confirmed its positive regulatory role in enhancing resistance to *A. alternata*.

## Data Availability

Publicly available datasets were analyzed in this study. This data can be found here: The whole genome data of broad beans: https://projects.au.dk/fabagenome/genomics-data Accession number of transcriptome sequencing data of broad beans: PRJNA1327605.

## Supplementary material

Supplementary Figure 1 Expression patterns of 45 *VfRLCK VII* genes in response to infection with *A. alternata*. The color bar in the upper right corner represented FPKM values based on log2 normalization, while blue to red indicated gene relative expression levels from low to high.
