# Supplementary figures and images for: Genome-wide identification and functional validation of RLCK VII subfamily genes conferring disease resistance in broad bean (Vicia faba L.)
Source: Front Plant Sci. 2026 Jan 21;16:1712686. doi: 10.3389/fpls.2025.1712686 (PMC12868224; doi:10.3389/fpls.2025.1712686)

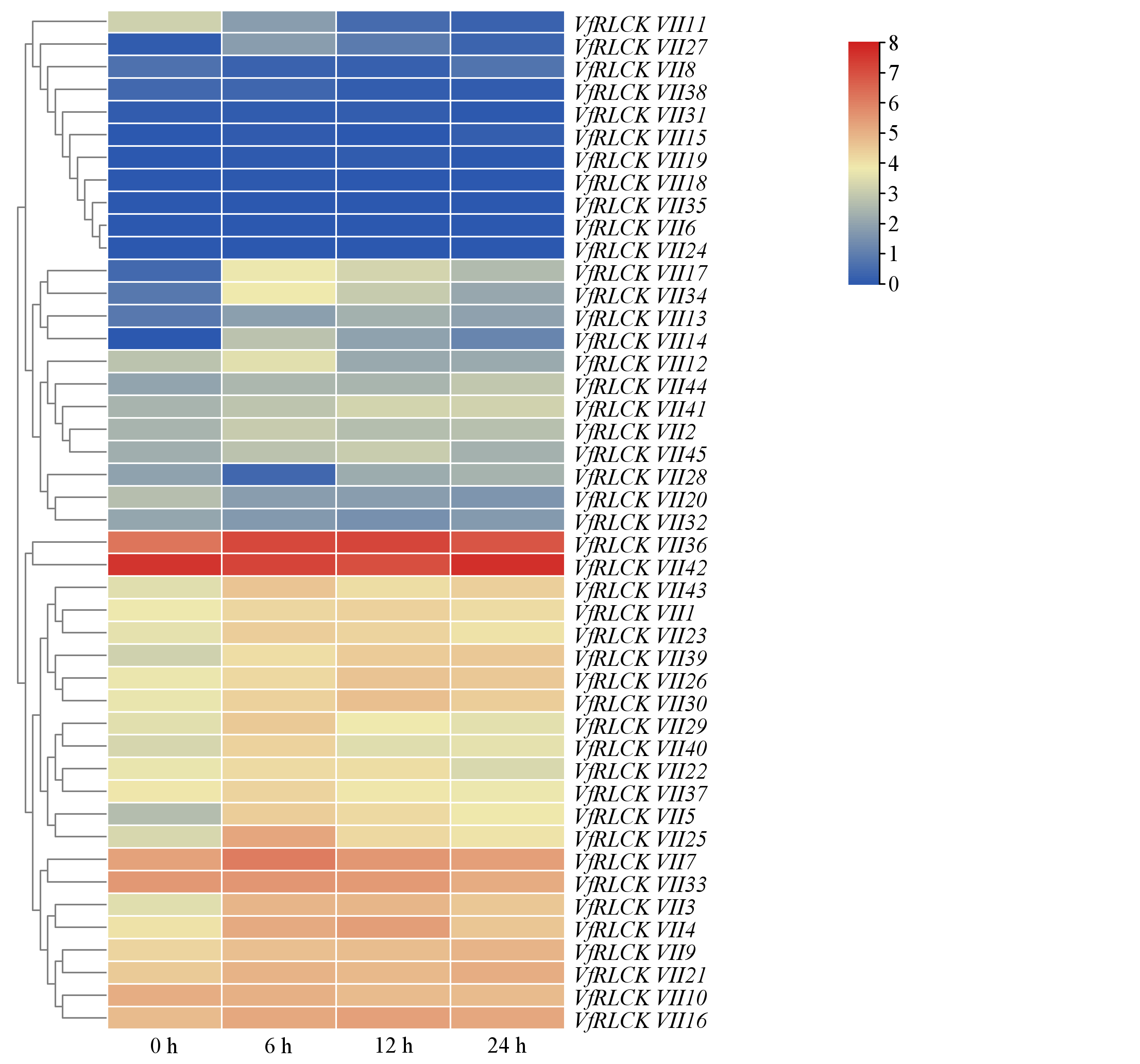

Supplement: Supplementary Figure 1 — Expression patterns of 45 VfRLCK VII genes in response to infection with A. alternata. The color bar in the upper right corner represented FPKM values based on log2 normalization, while blue to red indicated gene relative expression levels from low to high. [file Image1.tiff]
