# Supplementary material for: Genome-wide identification and functional validation of RLCK VII subfamily genes conferring disease resistance in broad bean (Vicia faba L.)
Source: Front Plant Sci. 2026 Jan 21;16:1712686. doi: 10.3389/fpls.2025.1712686 (PMC12868224; doi:10.3389/fpls.2025.1712686)
Supplement: Supplementary file 2 [file Table1.docx]

**Table S1. Primers used in this study**

| **Genes** | **Forward primers** | **Reverse primers** |
| --- | --- | --- |
| **qRT-PCR** | | |
| *VfRLCK VII3* | GCTGTATCATCTCATCAGTTCAAGG | AACAGGCTGACCCTTTTTTGG |
| *VfRLCK VII4* | ACCTTACCTCAACAGCAAACGG | GCCGTAACATCAGAGGAACCC |
| *VfRLCK VII5* | GCAATGCCTTTTCTGAGTGACA | ATCGGTTCCAGATTTTGGTGTC |
| *VfRLCK VII11* | CAAAAAGACATTCAGAGCAGCAG | ATAGAAGGGCGTTTTTTAGGGTC |
| *VfRLCK VII17* | CTTGAGTGATACCAGAAGAGTTTTGAG | AGAAGAGTGTTTGGTTGCTGGAG |
| *VfRLCK VII20* | TGGTGGATTTCACAAAGGGATA | CGCACTTCTCGGTTTTTTCAC |
| *VfRLCK VII25* | GCAATGAGTCAGGTGGTAGAGAGAT | GCCTCAACCGATTCATTTTCA |
| *VfRLCK VII36* | TTACCTATCCAACAAACGCAGAGT | CCTTTCTTCTGTGTATCTCTTGGTTC |
| *VfELF1A* | GTGAAGCCCGGTATGCTTGT | CTTGAGATCCTTGACTGCAACATT |
| *NtActin* | CCTGAGGTCCTTTTCCAACCA | GGATTCCGGCAGCTTCCATT |
| **gene cloning** | | |
| *VfRLCK VII4* | ATGGGTTGTTGCTTTAGTGCCAGAAT | TCATTTTTCATTGATTACTGTCTCAC |
| **vector construction** | | |
| *VfRLCK VII4* | AGAACACGGGGGACGAGCTCATGGGTTGTTGCTTTAGTGCCAGAA | ACCATGGTGTCGACTCTAGATTTTTCATTGATTACTGTCTCACTTTGTTTATTAC |
