# Supplementary material for: Genome-wide identification and functional validation of RLCK VII subfamily genes conferring disease resistance in broad bean (Vicia faba L.)
Source: Front Plant Sci. 2026 Jan 21;16:1712686. doi: 10.3389/fpls.2025.1712686 (PMC12868224; doi:10.3389/fpls.2025.1712686)
Supplement: Supplementary file 3 [file Table2.docx]

**Table S2. Physicochemical properties and subcellular localization of VfRLCK VII subfamily members**

| **Gene ID** | **Gene name** | **Amino acid number/aa** | **Molecular weight/Da** | **pI** | **Instability index** | **Fat index** | **Subcellular localization** |
| --- | --- | --- | --- | --- | --- | --- | --- |
| Vfaba.Tiffany.R1.1g022680.1 | *VfRLCK VII1* | 296 | 32530.79 | 9.45 | 23.58 | 80.10 | Nucleus |
| Vfaba.Tiffany.R1.1g025720.1 | *VfRLCK VII2* | 365 | 40579.89 | 9.09 | 28.42 | 76.38 | Nucleus |
| Vfaba.Tiffany.R1.1g104640.1 | *VfRLCK VII3* | 486 | 53848.57 | 9.62 | 34.97 | 75.43 | Nucleus |
| Vfaba.Tiffany.R1.1g192480.1 | *VfRLCK VII4* | 410 | 45339.36 | 9.39 | 42.93 | 77.78 | Chloroplast |
| Vfaba.Tiffany.R1.1g211440.1 | *VfRLCK VII5* | 395 | 43840.55 | 9.24 | 29.96 | 79.52 | Nucleus |
| Vfaba.Tiffany.R1.1g220320.1 | *VfRLCK VII6* | 374 | 41894.05 | 8.73 | 31.93 | 82.89 | Nucleus |
| Vfaba.Tiffany.R1.1g233440.1 | *VfRLCK VII7* | 391 | 42562.25 | 8.88 | 33.71 | 80.59 | Nucleus |
| Vfaba.Tiffany.R1.1g252240.1 | *VfRLCK VII8* | 361 | 40725.76 | 8.70 | 40.16 | 91.52 | Nucleus |
| Vfaba.Tiffany.R1.1g258080.1 | *VfRLCK VII9* | 492 | 54836.21 | 9.51 | 40.72 | 70.75 | Nucleus |
| Vfaba.Tiffany.R1.1g259120.1 | *VfRLCK VII10* | 410 | 44895.22 | 9.78 | 43.01 | 80.68 | Chloroplast |
| Vfaba.Tiffany.R1.1g282600.1 | *VfRLCK VII11* | 364 | 40642.89 | 9.24 | 31.89 | 85.74 | Nucleus |
| Vfaba.Tiffany.R1.1g308280.1 | *VfRLCK VII12* | 325 | 36320.87 | 9.64 | 29.53 | 87.91 | Nucleus |
| Vfaba.Tiffany.R1.1g308320.1 | *VfRLCK VII13* | 298 | 33903.24 | 8.14 | 26.48 | 85.07 | Nucleus |
| Vfaba.Tiffany.R1.1g334520.1 | *VfRLCK VII14* | 445 | 49900.74 | 9.61 | 45.93 | 72.7 | Nucleus |
| Vfaba.Tiffany.R1.1g371160.1 | *VfRLCK VII15* | 391 | 43373.39 | 6.28 | 28.69 | 84.35 | Nucleus |
| Vfaba.Tiffany.R1.1g376200.1 | *VfRLCK VII16* | 501 | 55521.47 | 9.01 | 34.92 | 65.63 | Nucleus |
| Vfaba.Tiffany.R1.2g024120.1 | *VfRLCK VII17* | 371 | 40632.16 | 9.43 | 31.99 | 84.64 | Nucleus |
| Vfaba.Tiffany.R1.2g033080.1 | *VfRLCK VII18* | 436 | 48955.19 | 4.63 | 34.07 | 74.93 | Nucleus |
| Vfaba.Tiffany.R1.2g143440.1 | *VfRLCK VII19* | 377 | 42295.76 | 8.72 | 33.59 | 96.45 | Nucleus |
| Vfaba.Tiffany.R1.2g170640.1 | *VfRLCK VII20* | 388 | 43921.56 | 9.55 | 28.49 | 82.65 | Nucleus |
| Vfaba.Tiffany.R1.2g170720.1 | *VfRLCK VII21* | 339 | 37602.45 | 8.85 | 39.02 | 90.59 | Nucleus |
| Vfaba.Tiffany.R1.3g032280.1 | *VfRLCK VII22* | 426 | 48136.97 | 9.4 | 40.26 | 82.14 | Nucleus |
| Vfaba.Tiffany.R1.3g043080.1 | *VfRLCK VII23* | 380 | 41909.16 | 9.30 | 30.66 | 84.45 | Nucleus |
| Vfaba.Tiffany.R1.3g065240.1 | *VfRLCK VII24* | 595 | 65018.03 | 8.19 | 45.32 | 56.27 | Nucleus |
| Vfaba.Tiffany.R1.4g004440.1 | *VfRLCK VII25* | 442 | 50142.75 | 8.1 | 39.91 | 77.62 | Nucleus |
| Vfaba.Tiffany.R1.4g005080.1 | *VfRLCK VII26* | 451 | 50265.95 | 9.08 | 44.08 | 71.35 | Nucleus |
| Vfaba.Tiffany.R1.4g046000.1 | *VfRLCK VII27* | 451 | 51000.72 | 9.36 | 37.85 | 90.31 | Nucleus |
| Vfaba.Tiffany.R1.4g065640.1 | *VfRLCK VII28* | 346 | 39367.19 | 7.69 | 44.02 | 83.41 | Nucleus |
| Vfaba.Tiffany.R1.4g082480.1 | *VfRLCK VII29* | 411 | 44871.15 | 9.34 | 42.42 | 80.22 | Nucleus |
| Vfaba.Tiffany.R1.4g104080.1 | *VfRLCK VII30* | 380 | 42901.85 | 9.35 | 33.61 | 77.47 | Nucleus |
| Vfaba.Tiffany.R1.4g122800.1 | *VfRLCK VII31* | 383 | 42142.11 | 9.45 | 35.33 | 87.13 | Nucleus |
| Vfaba.Tiffany.R1.4g135280.1 | *VfRLCK VII32* | 429 | 47824.51 | 9.32 | 36.28 | 78.41 | Chloroplast |
| Vfaba.Tiffany.R1.4g159080.1 | *VfRLCK VII33* | 494 | 55748.33 | 7.80 | 40.43 | 68.89 | Chloroplast |
| Vfaba.Tiffany.R1.4g162840.1 | *VfRLCK VII34* | 437 | 48388.61 | 9.67 | 27.5 | 82.59 | Nucleus |
| Vfaba.Tiffany.R1.5g026520.1 | *VfRLCK VII35* | 431 | 48637.83 | 8.15 | 39.02 | 79.63 | Nucleus |
| Vfaba.Tiffany.R1.5g037040.1 | *VfRLCK VII36* | 407 | 44946.69 | 9.67 | 37.11 | 74.57 | Chloroplast |
| Vfaba.Tiffany.R1.5g051280.1 | *VfRLCK VII37* | 441 | 48467.93 | 9.04 | 30.86 | 79.84 | Nucleus |
| Vfaba.Tiffany.R1.5g068880.1 | *VfRLCK VII38* | 424 | 47678.31 | 6.34 | 38.64 | 76.16 | Nucleus |
| Vfaba.Tiffany.R1.5g107840.1 | *VfRLCK VII39* | 379 | 42088.33 | 9.51 | 23.23 | 82.11 | Nucleus |
| Vfaba.Tiffany.R1.5g112040.1 | *VfRLCK VII40* | 426 | 48136.77 | 9.55 | 46.34 | 80.26 | Nucleus |
| Vfaba.Tiffany.R1.5g147960.1 | *VfRLCK VII41* | 590 | 64841.85 | 6.93 | 46.89 | 60.81 | Nucleus |
| Vfaba.Tiffany.R1.6g027840.1 | *VfRLCK VII42* | 410 | 45095.5 | 8.85 | 34.03 | 93.17 | Chloroplast |
| Vfaba.Tiffany.R1.6g027880.1 | *VfRLCK VII43* | 450 | 50829.37 | 7.72 | 42.44 | 82.29 | Nucleus |
| Vfaba.Tiffany.R1.6g107080.1 | *VfRLCK VII44* | 387 | 43579.92 | 7.19 | 26.23 | 86.93 | Nucleus |
| Vfaba.Tiffany.R1.6g139480.1 | *VfRLCK VII45* | 383 | 42652.59 | 8.20 | 32.96 | 84.05 | Nucleus |
