# Supplementary material for: Genome-wide identification and functional validation of RLCK VII subfamily genes conferring disease resistance in broad bean (Vicia faba L.)
Source: Front Plant Sci. 2026 Jan 21;16:1712686. doi: 10.3389/fpls.2025.1712686 (PMC12868224; doi:10.3389/fpls.2025.1712686)
Supplement: Supplementary file 4 [file Table3.docx]

**Table S3. *Ka*/*Ks* values of 18 fragment replication events of VfRLCK VII subfamily genes**

| **Gene ID** | **Gene ID** | ***Ka*** | ***Ks*** | ***Ka*/*Ks*** |
| --- | --- | --- | --- | --- |
| Vfaba.Tiffany.R1.1g258080.1 | Vfaba.Tiffany.R1.1g104640.1 | 0.186117276 | 3.227230008 | 0.057670905 |
| Vfaba.Tiffany.R1.1g233440.1 | Vfaba.Tiffany.R1.2g170640.1 | 0.331004931 | 2.501182655 | 0.132339368 |
| Vfaba.Tiffany.R1.1g282600.1 | Vfaba.Tiffany.R1.2g170640.1 | 0.342043526 | 1.748710641 | 0.195597555 |
| Vfaba.Tiffany.R1.1g282600.1 | Vfaba.Tiffany.R1.4g082480.1 | 0.175703452 | 0.937465128 | 0.187423986 |
| Vfaba.Tiffany.R1.1g308280.1 | Vfaba.Tiffany.R1.4g104080.1 | 0.140317965 | 1.258414257 | 0.111503795 |
| Vfaba.Tiffany.R1.1g334520.1 | Vfaba.Tiffany.R1.4g159080.1 | 0.201230533 | 1.503929817 | 0.133803141 |
| Vfaba.Tiffany.R1.1g258080.1 | Vfaba.Tiffany.R1.4g005080.1 | 0.088028092 | 0.75099052 | 0.117215982 |
| Vfaba.Tiffany.R1.1g334520.1 | Vfaba.Tiffany.R1.4g046000.1 | 0.353525886 | 1.411795186 | 0.250408763 |
| Vfaba.Tiffany.R1.1g259120.1 | Vfaba.Tiffany.R1.4g122800.1 | 0.22996743 | 2.081572538 | 0.11047774 |
| Vfaba.Tiffany.R1.1g252240.1 | Vfaba.Tiffany.R1.4g065640.1 | 0.080392884 | 0.764372038 | 0.105175072 |
| Vfaba.Tiffany.R1.1g104640.1 | Vfaba.Tiffany.R1.4g005080.1 | 0.193391116 | 1.746691083 | 0.110718557 |
| Vfaba.Tiffany.R1.1g022680.1 | Vfaba.Tiffany.R1.5g037040.1 | 0.259196047 | NaN | NaN |
| Vfaba.Tiffany.R1.2g024120.1 | Vfaba.Tiffany.R1.4g122800.1 | 0.33702609 | 1.852909099 | 0.181890245 |
| Vfaba.Tiffany.R1.2g170640.1 | Vfaba.Tiffany.R1.4g082480.1 | 0.359479246 | 2.576824033 | 0.139504771 |
| Vfaba.Tiffany.R1.3g032280.1 | Vfaba.Tiffany.R1.5g112040.1 | 0.130249825 | 0.839575744 | 0.15513767 |
| Vfaba.Tiffany.R1.3g043080.1 | Vfaba.Tiffany.R1.5g107840.1 | 0.094594066 | 0.635781435 | 0.148783939 |
| Vfaba.Tiffany.R1.4g046000.1 | Vfaba.Tiffany.R1.4g159080.1 | 0.337301929 | 1.463168093 | 0.230528488 |
| Vfaba.Tiffany.R1.5g037040.1 | Vfaba.Tiffany.R1.6g027840.2 | 0.276945611 | 2.95596567 | 0.093690402 |
