# Supplementary material for: Genome-wide identification and functional validation of RLCK VII subfamily genes conferring disease resistance in broad bean (Vicia faba L.)
Source: Front Plant Sci. 2026 Jan 21;16:1712686. doi: 10.3389/fpls.2025.1712686 (PMC12868224; doi:10.3389/fpls.2025.1712686)
Supplement: Supplementary file 6 [file Table5.docx]

**Table S5. A comparative functional annotation of RLCK VII proteins in broad beans and Arabidopsis**

| **VfRLCK VIIs** | **AtRLCK VIIs** | **Family subgroups** | **Functional annotation** |
| --- | --- | --- | --- |
| VfRLCK VII 16, VfRLCK VII 23, VfRLCK VII 39 | AtPBL27, AtPBS1 | RLCK VII-1 | chitin-induced immunity |
| VfRLCK VII 16, VfRLCK VII 23, VfRLCK VII 39 | AtPBL7 | RLCK VII-1 | brassinosteroid signaling and plant growth |
| VfRLCK VII35 | AtPBL24, AtPBL25, AtPBL26 | RLCK VII-2 | fertilization specifically pollen tube burst within the synergid |
| VfRLCK VII22, VfRLCK VII 40 | AtPBL39 (PCRK1), 40 (PCRK2) | RLCK VII-4 | pattern-triggered immunity |
| VfRLCK VII12, VfRLCK VII25, VfRLCK VII30 | AtPBL19 | RLCK VII-4 | PRR-mediated resistance |
| VfRLCK VII9, VfRLCK VII26 | AtPBL34, AtPBL35, AtPBL36 | RLCK VII-5 | root meristem homeostasis |
| VfRLCK VII14, VfRLCK VII 33 | AtPBL14 (RIPK) | RLCK VII-6 | activation of a plant innate immune receptor RPM1 |
| VfRLCK VII11, VfRLCK VII29 | AtPBL30 | RLCK VII-7 | a spatial inhibitor of cell separation |
| VfRLCK VII4 | AtPBL1 and AtBIK1 | RLCK VII-8 | resistance responses to pathogen, leaf morphogenesis and inflorescence architecture |
| VfRLCK VII38 | AtCDG1 |  | abnormal differential and elongation growth after organ differentiation |
